# The ^40^Ar/^39^Ar dating of quartz: new insights into the metallogenic chronology of the Jinchang gold deposit and its geological significance

**DOI:** 10.1038/s41598-018-32242-3

**Published:** 2018-09-17

**Authors:** Kaituo Shi, Keyong Wang, Haijun Yu, Zhigao Wang, Xueli Ma, Xiujuan Bai, Rui Wang

**Affiliations:** 1 College of Earth Sciences, Jilin University, Changchun, 130061 China; 2 MLR Key Laboratory of Sanjiang Metallogeny and Resources Exploration and Utilization, Yunnan Geological Survey, Kunming, 650051 China; 3 MOE Key Laboratory of Tectonics and Petroleum Resources, China University of Geosciences, Wuhan, 430074 China; 4 School of Prospecting and Surveying Engineering, Changchun Institute of Technology, Changchun, 130061 China

## Abstract

The Jinchang gold deposit has been extensively studied, but precise dates for its formation are debated. Native gold mainly occurs as inclusions within pyrite and quartz. In this study, we analysed quartz crystals coeval with gold precipitation from two different types of mineralization using the ArgusVI multi-collector noble gas mass spectrometer by the stepwise crushing technique to resolve the timing and genesis of gold mineralization. ^40^Ar/^39^Ar dating of quartz samples (J12Q) from breccia ore yields a plateau age of 109.87 ± 0.86 Ma, and an inverse isochron age of 109.87 ± 0.88 Ma. Quartz samples (J18Q) from vein ore yields a slightly younger plateau age of 107.76 ± 0.85 Ma, with an inverse isochron age of 107.76 ± 0.92 Ma. These dates place the ore-forming age of the Jinchang gold deposit at 107~110 Ma, much younger than previously published radiometric ages, suggesting the gold mineralization is spatio-temporally associated with the granite porphyry. The formation of the Jinchang gold deposit is consistent with the regional late Mesozoic porphyry-epithermal gold mineralization event in the Yanbian-Dongning area. Finally, our study shows that ^40^Ar/^39^Ar of quartz can be used as a powerful tool to date the formation ages of hydrothermal ore deposits.

## Introduction

Studies on the genesis of hydrothermal gold mineralization are often hampered by a lack of metallogenic age information because minerals suitable for conventional radiometric dating are not always available and diverse dating methods have their own limitations^1^. In many cases, the timing of gold precipitation has to be inferred by dating magmatic, deformation, metamorphic, and/or alteration events using traditional methods^2^. However, such a dating procedure cannot be applied to gold deposits that record multistage tectonothermal events.

The accurate age of the mineralization also plays a pivotal role in summarizing metallogenic regularity, establishing a metallogenic model, and guiding further exploration. Thus, a precise dating method is required in order to determine which magmatic, tectonic, and/or metamorphic events in the vicinity of the mineralization, if any, are genetically related to the gold mineralization. With the development of analytical techniques and a new generation of mass spectrometers, more attention has been focused on the ^40^Ar/^39^Ar stepwise crushing technique, which has been applied to successfully date the time of ore formation of lead-zinc and tungsten deposits recently^3–5^. These cases show the potential of this method for determining the ore-forming time of hydrothermal mineral deposits.

**Figure 1:**
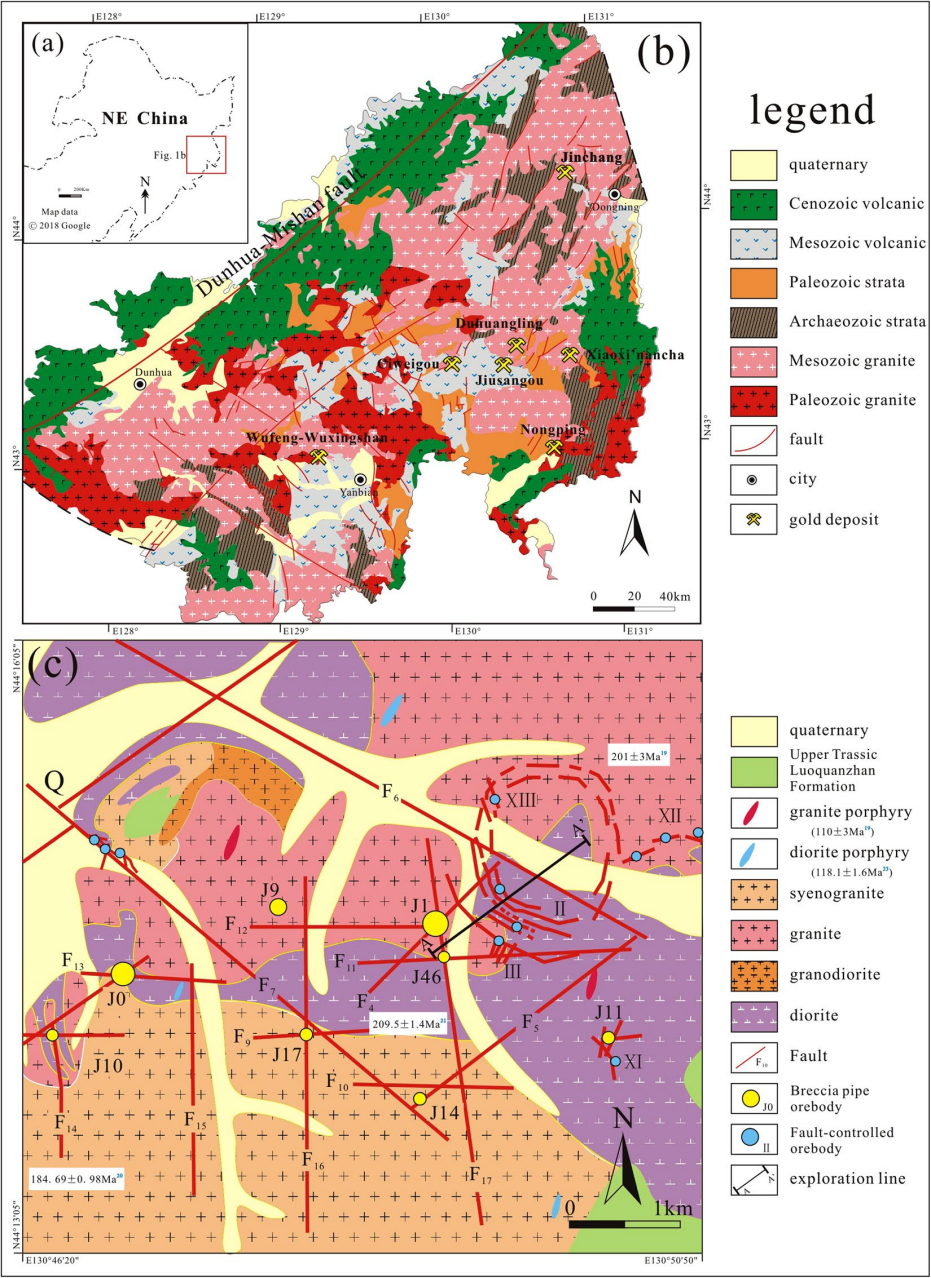
Maps showing the location (**a**) and general geology (**b**) of the Yanbian-Dongning gold mineralization belt; (**c**) geologic sketch map of the Jinchang gold deposit.

The Yanbian-Dongning area along the southeastern margin of NE China is a major gold producing region with a complex tectonothermal history. The region experienced the closure of the Paleo-Asian Ocean in the Paleozoic and subduction of the Paleo-Pacific plate in the Mesozoic^6–10^. Furthermore, previous geochronological studies indicated that gold mineralization around the Yanbian-Dongning area of NE China (e.g., the Xiaoxi’nancha, Duhuangling, Jiusangou, Ciweigou and Wufeng-Wuxingshan) is mainly Mesozoic and clustered at 100~110 Ma^11–15^. The Jinchang gold deposit, located in the northeastern part of the Yanbian-Dongning area (Fig. 1b), differs from other gold deposits in this region by its distinctive types of mineralization, which include cryptoexplosive breccia pipe type, veinlet disseminated type, and fault-controlled veins from early to late estimated by the occurrences of ore bodies as well as decreasing compositional complexity and homogenization temperature of fluid inclusions^16^. Despite the various methods used to date the absolute age of the Jinchang gold deposit, no consensus has yet been reached. Qing *et al*.^17^ obtained a ^40^Ar/^39^Ar isochron age of 124 ± 6 Ma (MSWD = 14) for a sphalerite sample from the No. J-9 cryptoexplosive breccia pipe orebody using the single grain Ar-Ar laser probe method. A Re-Os isochron age of 114 ± 22 Ma (MSWD = 0.15) was reported for five pyrites samples from the No. J-1 cryptoexplosive breccia pipe orebody^18^.

These scattered and imprecise dates suggest that the following questions remain unresolved: (1) the absolute time of various types of hydrothermal gold mineralization in this deposit, (2) which tectonomagmatic activity is associated with the Jinchang gold deposit, and (3) whether the age and setting of the Jinchang gold deposit are identical to those of the other widely distributed gold deposits around the Yanbian-Dongning area or not. Therefore, quartz that formed coevally with the gold from the cryptoexplosive breccia pipe orebody (early) and fault-controlled vein type orebody (late) were sampled and selected for ^40^Ar/^39^Ar stepwise crushing to precisely constrain the absolute timing of hydrothermal gold mineralization in the Jinchang gold deposit and discuss its significance for the geodynamic evolution and regional metallogeny in the Yanbian-Dongning area.

## Geological setting

**Figure 2:**
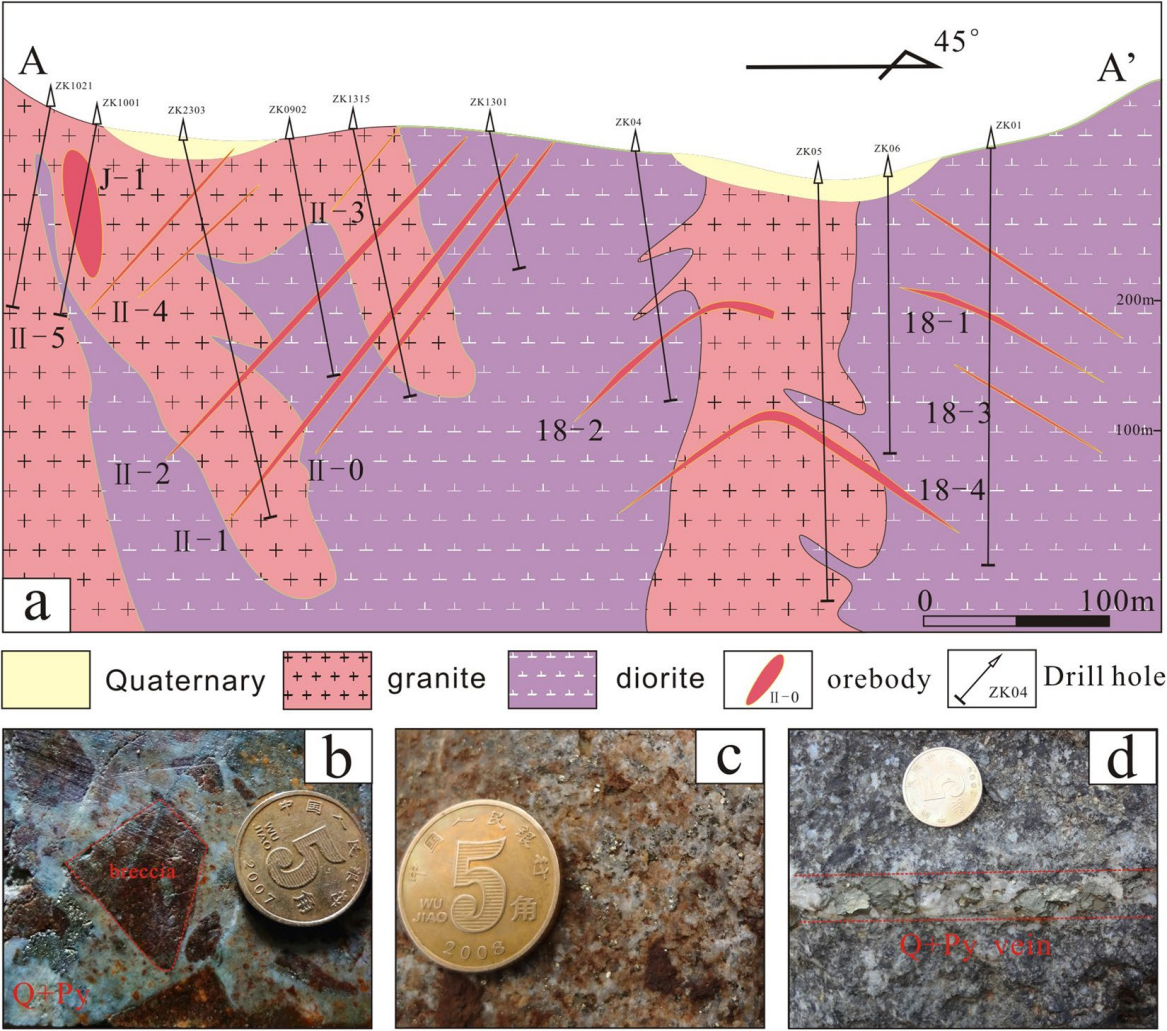
(**a**)Geologic cross section showing three types of mineralization (After Zhao^21^); (**b**) breccia ore; (**c**) disseminated ore; (**d**) vein ore. Abbreviations: Q = quarta, Py = pyrite.

The giant Jinchang gold deposit (>2.57 Moz Au) is located in the northeastern part of the Yanbian-Dongning gold mineralization belt, which is well-endowed with epithermal and porphyry gold deposits and is an important area of gold mineralization in NE China (Fig. 1a,b). The Jinchang mine is a famous gold-rich porphyry deposit with three types of mineralization, namely cryptoexplosive breccia pipe type (No. J-0, J-1, J-2, J-9, J-10, J-17, J-46 pipe), veinlet disseminated type (No. J-18 ore body), as well as ring and radial fault-controlled veins (No. II, X, XI and No. III, VIII, VII vein swarms) (Figs 1, 2), among which the first type is of greatest importance, hosting >41% of the total gold resource^19^. Most of the gold ore is associated with quartz aggregates, veins, or veinlets (Fig. 2b–d).

The main lithostratigraphic units in the Jinchang area are the upper Triassic Luoquanzhan Formation, which is composed of rhyolitic-dacitic tuff and scattered in the southeastern part of the deposit (Fig. 1c). Mineralization in the district is predominantly hosted by the widespread early Mesozoic (185~210 Ma)^19–21^ granite, diorite, granodiorite and syenogranite (Figs 1c, 2). However, the locally distributed late Mesozoic (110~120 Ma) granite porphyry and diorite porphyry were thought to be temporally and genetically associated with the gold mineralization^18–20,22,23^. Structurally, the ore district is characterized by widely developed NE-, NW-, N-, and E-trending linear fractures with subordinate ring and radial faults (Fig. 1c). The cryptoexplosive breccia pipes occur in the intersections of variously oriented linear faults, whereas ring and radial fractures control the occurrence of vein orebodies. The principal metallic minerals are pyrite and chalcopyrite, with minor amounts of galena, sphalerite, molybdenite, and magnetite. Gangue minerals consist of quartz, calcite, feldspar, sericite, chlorite, epidote, and kaolinite. Native gold mainly occurs as inclusions within pyrite and quartz^21^. New findings indicate some siegenite and tetradymite also host native gold^24,25^. Rocks within the Jinchang deposit record intense hydrothermal alteration which includes potassic alteration, silicification, sericitization, chloritization, kaolinization, and carbonatization, among which silicification and sericitization show close spatial relationships with the gold mineralization^18,19^. The alteration characteristics are also comparable to the observations of porphyry-type system^26^.

**Figure 3:**
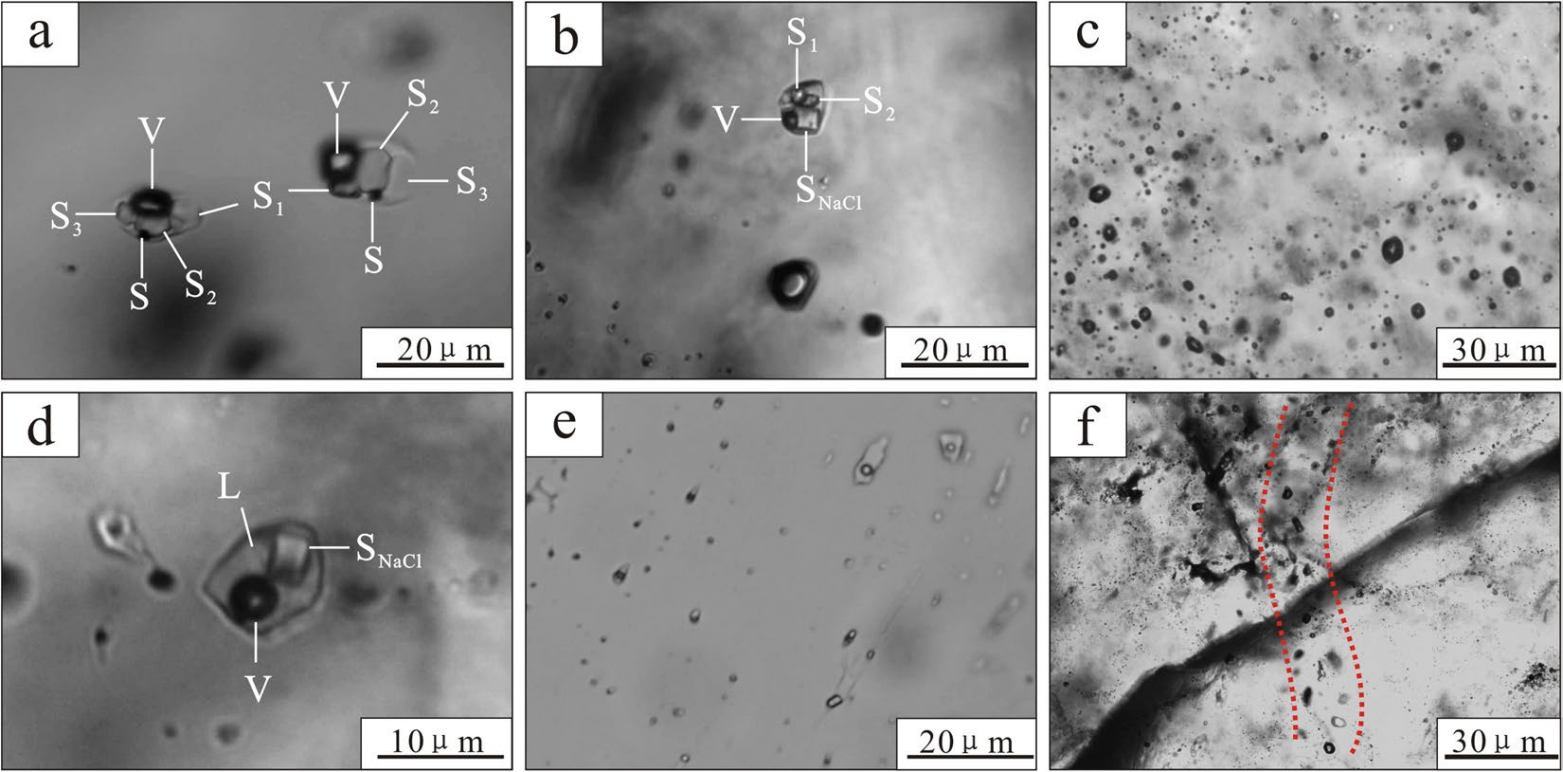
Photomicrographs of representative fuid inclusion types at room temperature in the auriferous quartz samples. (**a**,**b**) Daughter mineral-bearing multi-phase FIs; (**c**) vapor-rich two-phase FIs; (**d**) halite daughter mineral-bearing three-phase FIs; (**e**) aqueous two-phase FIs; (**f**) secondary fluid inclusions. Abbreviations: L = liquid; V = vapor; S = metal sulfide; S_1_, S_2_, S_3_ = K-rich silicate; S_NaCl_ = halite.

The auriferous quartz samples from three types of mineralization contain abundant primary fluid inclusions (FIs), which consist of daughter mineral-bearing multi-phase FIs (number of daughter minerals ≥4), vapor-rich two-phase FIs, halite daughter mineral-bearing three-phase FIs, and aqueous two-phase FIs (Fig. 3a–e). Minor amounts of secondary fluid inclusions, which are aligned along the micro-fractures in transgranular trails, are also present (Fig. 3f). Previous microthermometry results indicate that the ore-forming fluid belongs to a moderate-high temperature (>400 °C) and high salinity (>40 wt.% NaCl eqv.) NaCl-H_2_O system^16,27^. The compositions of daughter minerals are remarkably complex, including opaque pyrite, chalcopyrite, sphalerite, and transparent halite, sylvite, K-rich silicate, determined by SEM/EDS analysis^16^.

## Sampling Technique

Quartz samples from No. J-1 pipe breccia ore (J12Q) and No. II vein ore (J18Q) were selected for ^40^Ar/^39^Ar dating using the stepwise crushing methods. Single quartz grains were crushed and sieved into 30–60 mesh (0.50–0.25 mm), then hand-picked under a binocular microscope and cleaned in an ultrasonic bath with deionized water for 15 min.

## Results

**Figure 4:**
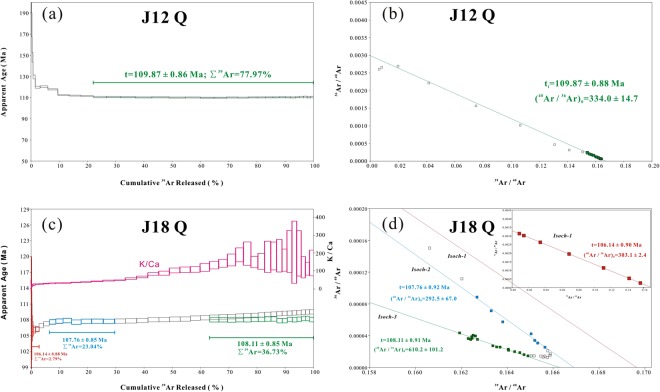
Plots of ^40^Ar/^39^Ar age spectra and inverse isochron lines of quartz samples J12Q and J18Q.

The ^40^Ar/^39^Ar dating results of quartz samples are displayed in Fig. 4. The argon isotope intensities and their uncertainties are expressed throughout in fA. The calculated ages are reported at the 2σ level in this paper.

The J12Q sample was crushed in 34 steps with a total number of 19440 pestle drops. The apparent ages decrease dramatically during the first 11 steps, followed by a flat plateau. On the inverse isochron diagram of ^36^Ar/^40^Ar vs. ^39^Ar/^40^Ar, the data points of steps 12–34 define an excellent linear array, and yield an age of 109.87 ± 0.88 Ma (MSWD = 0.43), with an initial ^40^Ar/^36^Ar ratio of 334.0 ± 14.7, which is slightly higher than the modern atmosphere ratio of 298.56^28^. This means that some excess ^40^Ar was trapped in the fluid inclusions, which is supported by the topology of the age spectrum. The excess ^40^Ar affects the plateau part of the age spectrum. By applying the ratio of 334.0 to exclude non-radiogenic ^40^Ar, a flat plateau of 109.87 ± 0.86 Ma (MSWD = 0.41) is obtained, with 77.97% ^39^Ar released (Fig. 4a, dashed green lines). The correction is made with the assumption that the excess ^40^Ar is homogeneously distributed in those regions of the lattice that degassed to form the plateau on the age spectrum.

The J18Q sample was crushed in 42 steps with a total of 23110 pestle drops. On the inverse isochron diagram, the crushing data points form three well-defined isochron lines (Fig. 4d). The first seven steps define a concordant isochron line with age of 106.14 ± 0.90 Ma (MSWD = 0.12) corresponding to an initial ^40^Ar/^36^Ar ratio of 303.1 ± 2.4; the data points of steps 10–16 define an isochron corresponding to 107.76 ± 0.92 Ma (MSWD = 0.29), with the initial ^40^Ar/^36^Ar value of 292.5 ± 67.0; the last seventeen steps yield a well-defined isochron line with age of 108.11 ± 0.91 Ma (MSWD = 0.32), corresponding to an initial ^40^Ar/^36^Ar ratio of 610.2 ± 101.2. By applying the initial ^40^Ar/^36^Ar ratios to exclude non-radiogenic ^40^Ar, three flat plateau are obtained, which are 106.14 ± 0.88 Ma (MSWD = 0.10, Σ^39^Ar = 2.79%), 107.76 ± 0.85 Ma (MSWD = 0.24, Σ^39^Ar = 23.04%), and 108.11 ± 0.85 Ma (MSWD = 0.30, Σ^39^Ar = 36.73%), in concordance with their isochron ages respectively. Notably, the excess ^40^Ar correction is applied to isochron-3 with the assumption that the excess ^40^Ar is homogeneously distributed in those regions of the lattice that degassed to form the plateau on the age spectrum. The first group is interpreted as originating from contributions of the secondary fluid inclusions (SFIs), with K/Ca ratios less than 25, because the SFIs distributing along the microcracks are easily released by crushing. The second group (K/Ca = 30~45) is interpreted as the contributions from primary fluid inclusions (PFIs), representing the ore formation age. The last group (K/Ca > 110) represents a mixture from K-rich daughter minerals in PFIs and K-rich microlites within quartz grains.

## Discussion

### Age and genesis of the Jinchang deposit

Quartz was chosen for determining the metallogenic age in the Jinchang deposit by ^40^Ar/^39^Ar stepwise crushing technique because of the following reasons: (1) previous studies indicated that native gold mainly occurs as inclusions within quartz and pyrite^21^, and the presence of sulfide daughter minerals-bearing FIs in the quartz further suggests the mineralization and quartz are contemporaneous (Fig. 3a); (2) quartz itself has low radioactivity after irradiation^29^; (3) it has an abundance of K-rich minerals-bearing FIs (as noted above and verified by the analysis results of high concentration of ^39^Ar_K_) (Fig. 3a,b) and has high chemical purity in its lattice^30^, thus neutron induced noble gas isotopes released during analysis are predominantly from the fluid inclusions and not the lattice. Assuming the fluid inclusions, quartz crystals, and gold grains are syngenetic, the Ar-Ar dates reported in this study can be taken as the age of ore formation in the Jinchang gold deposit.

The J12Q sample from orebody No. J-1 yields identical inverse isochron and plateau ages which suggest that 109.87 ± 0.88 Ma can be regarded as the ore-forming age of the cryptoexplosive breccia pipe type gold mineralization. The J18Q sample from ore vein No. II yields slightly younger inverse isochron and plateau ages, thus 107.76 ± 0.92 Ma can stand for the ore-forming age of fault-controlled vein gold mineralization. The dating results from this study reveal that breccia type gold mineralization formed earlier than fault-controlled vein mineralization in the Jinchang deposit, which coincides with previous estimates based on occurrences of ore bodies as well as decreasing compositional complexity and homogenization temperature of fluid inclusions^16^. Thus, we conclude the absolute timing of gold mineralization at Jinchang appears to be 107~110 Ma. In contrast, previous determinations of the timing of gold mineralization are older than our results^17,18^. Qing *et al*.^17^ obtained a ^40^Ar/^39^Ar isochron age of 124 ± 6 Ma (MSWD = 14) for a sphalerite sample in the breccia ore using single grain Ar-Ar laser probe method. The possible reason for the conflicting geochronological data include the ambiguous relationships between gold and sphalerite, and some excess ^40^Ar trapped during sphalerite crystallization. Zhang *et al*.^18^ reported a Re-Os isochron age of 114 ± 22 Ma (MSWD = 0.15) for five pyrites samples from the No. J-1 pipe orebody, scattered and less precise than our results. We speculate that the Re-Os isotopic system in pyrite may be disturbed by later tectonism and hydrothermal fluid. Considering that hydrothermal quartz is a ubiquitous gangue constituent in hydrothermal deposits, our study further illustrates the applicability of the quartz ^40^Ar/^39^Ar stepwise crushing technique to the direct dating of hydrothermal ore deposits.

The H-O-S-Pb isotopic compositions^21^, together with widespread melt-fluid inclusions and sulfide daughter minerals-bearing FIs in the auriferous quartz samples (Fig. 3a,b) from Jinchang deposit^16^, indicate the ore metals and fluids came primarily from a magmatic source. Previous geochronological studies of intrusive rocks in the Jinchang deposit have shown that at least two periods of magmatic activity have been recognized in the mining region, including widely outcropping early Mesozoic (185~210 Ma)^19–21^ granite, diorite, granodiorite and syenogranite, as well as the locally distributed late Mesozoic (110~120 Ma)^19,20,23^ granite porphyry and diorite porphyry. Among them, the granite porphyry records intense phyllic alteration, accompanied by abundant mineralization^19^. The emplacement age of the granite porphyry has been well constrained, concentrated at 110~113 Ma^19,20^ which is broadly consistent with the time of the gold mineralization. We therefore infer that the gold mineralization is spatio-temporally and genetically associated with the granite porphyry. The emplacement of the granite porphyry and subsequent pulses of volatile release and gas streaming resulted in breccia pipes and fractures. When the ore-bearing fluids exsolved from the granite porphyry, they migrated into the breccia pipes and fractures, leading to the intense alteration and precipitating abundant ore minerals due to water-rock reactions and change of physicochemical conditions.

### Timing of the regional gold mineralization and tectonic implications

**Figure 5:**
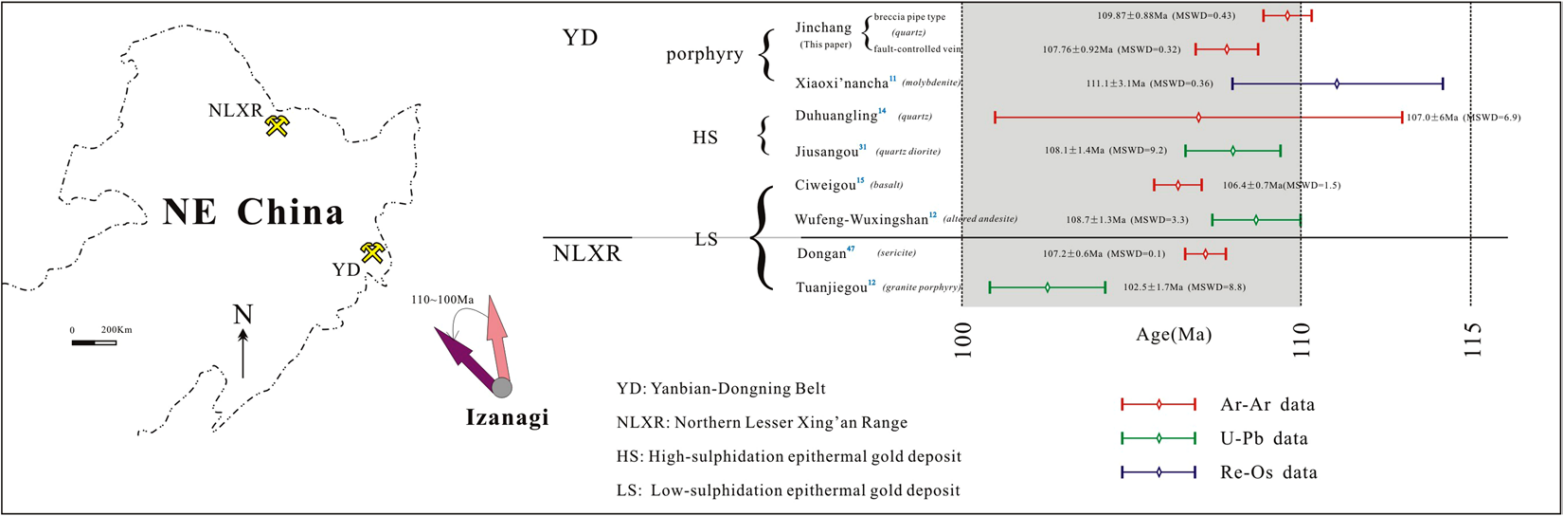
Published radiometric ages of the porphyry and epithermal gold deposits along eastern margin of NE China.

The Yanbian-Dongning region is an important area of porphyry-epithermal gold mineralization in NE China. To date, three gold-rich porphyry deposits (Xiaoxi’nancha, Jinchang, and Nongping), two high-sulfidation (HS) epithermal deposits (Duhuangling and Jiusangou), and two low-sulfidation (LS) epithermal deposits (Ciweigou and Wufeng-Wuxingshan) have been discovered (Fig. 1b). Recent studies reveal that the gold mineralization occurred throughout the Yanbian-Dongning region in the late Mesozoic around 100~110 Ma (Fig. 5). The Re-Os date of molybdenite from the Xiaoxi’nancha deposit is 111.1 ± 3.1 Ma^11^; the Nongping deposit has a sericite ^40^Ar/^39^Ar date of 95.0 ± 3.1 Ma^13^; a sulfide-bearing quartz vein from the Duhuangling deposit yielded a ^40^Ar/^39^Ar isochron age of 107 ± 6 Ma by laser probe techniques^14^; in the Jiusangou deposit, metallogenic porphyritic quartz diorite yielded a zircon U-Pb age of 108.1 ± 1.4 Ma^31^; Sun *et al*.^12^ considered the Ciweigou and Wufeng-Wuxingshan deposits formed after 108 Ma based on the ages of mineralized intrusives and host volcanic rocks. This study reveals that the Jinchang deposit belongs to this important gold mineralization event in the Yanbian-Dongning region, with metallogenic age of 107~110 Ma. Interestingly, the porphyry deposits (Xiaoxi’nancha and Jinchang; 108~111 Ma) formed earlier than epithermal deposits (~108 Ma), and the HS epithermal deposits are geographically located between the porphyry deposits and LS epithermal deposits (Fig. 1b). These observations are similar to those applicable to the typical porphyry-epithermal system^26^, such as the Zijinshan mining district in the SE China^32^, suggesting the formation of aforementioned deposits in the Yanbian-Dongning region shares a similar tectonic background.

The Yanbian-Dongning region, located in the southeastern margin of NE China, is recognized as a unique zone that underwent two stages of evolution within the tectonic regimes of the Paleo-Asian and Paleo-Pacific oceans^6–8^. Although the transformation time of the two tectonic regimes is still debated, it is generally accepted that the study area was associated with subduction of the Paleo-Pacific plate (Izanagi plate) during the Mesozoic^7,33,34^. The temporal–spatial distribution of Mesozoic granitoids and volcanic rocks in the Yanbian area demonstrates that an arc front migrated oceanwards, probably as a result of roll-back of the subducted Pacific plate (Izanagi plate)^9,35,36^. During the late Mesozoic, the presence of early Cretaceous A-type granite and alkali rhyolite^37^, metamorphic core complexes^38,39^, typical bimodal igneous rock association^40^, and the development of extensional basin (Songliao Basin)^41^ suggest that the whole NE China was in an extensional setting. The gold deposits in the Yanbian-Dongning region probably formed in such an extensional regime induced by the roll-back of the Paleo-Pacific plate (Izanagi plate).

The specific tectonic settings of particular metallogenic systems can be used as a potential criterion of geodynamic background. Many studies ascribe the large-scale Jiaodong-type gold mineralization (120 ± 5 Ma) to the changes in drift direction of the Pacific plate^42–44^; Choi *et al*.^45^ considered that a major shift in Izanagi plate dynamics at ca. 160 Ma led to the Middle to Late Jurassic gold event in the Korean Peninsula; during the 110~100 Ma, the subduction direction of the Izanagi plate changed from NNW to NW^46^, which may have played an important role in forming the porphyry-epithermal gold mineralization in the Yanbian-Dongning region. The contemporaneous gold systems along the west Pacific margin in the the Korean Peninsula, Northern Lesser Xing’an Range of NE China (Dongan and Tuanjiegou)^12,47^, as well as the Okhotsk–Chukotka belt and the Sikhote–Alin orogen of Far East Russia, may also have formed under such plate shift regime.

## Methods

The quartz samples were packed in aluminium foil and loaded into aluminium tubes together with the flux monitor ZBH-2506 biotite (132.7 ± 0.1 Ma, 1σ)^48^ that was packed in copper foil. The tubes were then irradiated for 48 h in the China Mianyang Research Reactor (CMRR).

The argon isotope analyses were undertaken in the Key Laboratory of Tectonics and Petroleum Resources, China University of Geosciences, Wuhan, China, using a new generation multi-collector Thermo Scientific ArgusVI mass spectrometer. The fluid inclusions were extracted in a crushing apparatus linked to the purification system and mass spectrometer. Details of the instruments are documented in Qiu *et al*.^49^. Prior to experiments, the extraction and purification systems are baked out with heat tapes, and sample in the tube is heated to 150 °C with a furnace for ~10 h to reduce system blanks. Cool blanks were carried out at the start and end of each sample experiment, and between every four to six steps of sample analyses. Samples were crushed by repeatedly lifting and dropping the pestle until the argon level diminished significantly, indicating exhaustion of the source of argon in the fluid inclusions. As the gas releases were decreased with progressive extraction steps, the number of pestle drops for each successive step was increased to maintain argon levels that could be measured precisely. The released gases were first cleaned through the cryotrap (−100 °C) to absorb the moisture from fluid inclusions, then further purified using three SAES NP10 Zr/Al getters (two at room temperature and one at ~400 °C), resulting in purified noble gases for argon isotope analyses in the mass spectrometer. For the current study, ^36^Ar was measured using a Compact Discrete Dynode (CDD) detector, with the remaining argon isotopes measured on Faraday detectors (H1, AX, L1, L2).

Full raw data are provided, and have been corrected for baselines, detector intercalibration and discrimination, radioactive decay, nucleogenic interferences, and backgrounds. Detector intercalibration and discrimination corrections were made using measurements of atmospheric argon and assuming a ^40^Ar/^36^Ar value of 298.56 ± 0.31^28^. Correction factors for interfering argon isotopes derived from irradiated CaF_2_ and K_2_SO_4_ are (^39^Ar/^37^Ar)_Ca_ = 6.175 × 10^−4^, (^36^Ar/^37^Ar)_Ca_ = 2.348 × 10^−4^ and (^40^Ar/^39^Ar)_K_ = 2.32 × 10^−3^. The ^40^Ar/^39^Ar dating results were calculated and plotted using the software ArArCALC (Version 2.52)^50^.

## Electronic supplementary material

Dataset 1

## References

[1] Chen W (2011). Isotope Geochronology: Technique and Application. Acta Geologica Sinica..

[2] Angln CD, Jonasson IR, Franklin JM (1996). Sm-Nd Dating of Scheelite and Tourmaline: Implications for the Genesis of Archcan Gold Deposits, Val d’Or, Canada. Econ Geol..

[3] Qiu H, Jiang Y (2007). Sphalerite ^40^Ar/^39^Ar Progressive Crushing and Stepwise Heating Techniques. Earth Planet Sc Lett..

[4] Jiang Y, Qiu H, Xu Y (2012). Hydrothermal Fluids, Argon Isotopes and Mineralization Ages of the Fankou Pb–Zn Deposit in South China: Insights From Sphalerite ^40^Ar/^39^Ar Progressive Crushing. Geochim Cosmochim Ac..

[5] Bai X, Wang M, Jiang Y, Qiu H (2013). Direct Dating of Tin–Tungsten Mineralization of the Piaotang Tungsten Deposit, South China, by ^40^Ar/^39^Ar Progressive Crushing. Geochim Cosmochim Ac..

[6] Jia D, Hu R, Lu Y, Qiu X (2004). Collision Belt Between the Khanka Block and the North China Block in the Yanbian Region, Northeast China. J Asian Earth Sci..

[7] Wu F (2011). Geochronology of the Phanerozoic Granitoids in Northeastern China. J Asian Earth Sci..

[8] Guo F (2015). Early Jurassic Subduction of the Paleo-Pacific Ocean in NE China: Petrologic and Geochemical Evidence From the Tumen Mafic Intrusive Complex. Lithos..

[9] Ma X, Zhu W, Zhou Z, Qiao S (2017). Transformation From Paleo-Asian Ocean Closure to Paleo-Pacific Subduction: New Constraints From Granitoids in the Eastern Jilin–Heilongjiang Belt, NE China. J Asian Earth Sci..

[10] Yang D, Sun D, Gou J, Hou X (2017). U–Pb Ages of Zircons From Mesozoic Intrusive Rocks in the Yanbian Area, Jilin Province, NE China: Transition of the Paleo-Asian Oceanic Regime to the circum-Pacific Tectonic Regime. J Asian Earth Sci..

[11] Ren Y, Wang H, Qu W, Zhao H, Chu G (2011). Re-Os Isotopic Dating of Molybdenite from Xiaoxinancha Copper-Gold Deposit in the Yanbian Area and its Geological Significance. Earth Science..

[12] Sun J (2013). Timing of Formation and Geological Setting of Low-Sulphidation Epithermal Gold Deposits in the Continental Margin of NE China. Int Geol Rev..

[13] Han S (2013). Geology and Ages of Porphyry and Medium- to High-Sulphidation Epithermal Gold Deposits of the Continental Margin of Northeast China. Int Geol Rev..

[14] Chai P, Sun J, Xing S, Men L, Han J (2014). Early Cretaceous Arc Magmatism and High-Sulphidation Epithermal Porphyry Cu–Au Mineralization in Yanbian Area, Northeast China: The Duhuangling Example. Int Geol Rev..

[15] Li, C. *Petrogenesis and Geological Implications of the Late Mesozoic Volcanic Rocks in Southeastern Jilin Province*, *Northeastern China*: Guangzhou Institute of geochemistry, Chinese Academy of Sciences (2006).

[16] Wang K, Qing M, Zhang X, Wan D, Xiao L (2011). Study On the Characteristics of Fluid Inclusions and Metallogenic Evolution of Jinchang Gold Deposit, Heilongjiang Province. Acta Petrol Sin..

[17] Qing M, Tang M, Xiao L, Zhao Y, Han X (2012). Automatic LaserProbe ^40^Ar/^39^Ar Isochron Ages of the Quatrz and Sphalerite From the Jinchang Gold Deposit, Dongning County, Heilongjiang Province and their Prospecting Implications. Geology and Exploration..

[18] Zhang P (2016). Re-Os Isotopic and Trace Element Compositions of Pyrite and Origin of the Cretaceous Jinchang Porphyry Cu-Au Deposit, Heilongjiang Province, NE China. J Asian Earth Sci..

[19] Zhang H (2013). Magmatism and Metallogeny Associated with Mantle Upwelling: Zircon U-Pb and Lu-Hf Constraints From the Gold-Mineralized Jinchang Granite, NE China. Ore Geol Rev..

[20] Han SJ (2018). U-Pb Zircon and Geochemical Constraints On Age and Genesis of Granitoids From the Jinchang Au Deposit in Heilongjiang, NE China. Geol J..

[21] Zhao, Y. Porphyry Gold System of the Jinchang Camp in the Yanbian-Dongning Metallogenic Belt, NE China: China University of Geosciences. (Beijing, 2013).

[22] Yu B, Zeng Q, Wang Y, He H, Su F (2017). The Sources of Ore-Forming Fluids from the Jinchang Gold Deposit, Heilongjiang Province, NE China: Constraints from the He-Ar Isotopic Evidence. Resour Geol..

[23] Zhao Y (2012). Geochemisry and Zircon U-Pb Geochronology of the Diorite Porphyry Associated with the Jinchang Cu-Au Deposit, Heilongjiang Province. Acta Petrol Sin..

[24] Zhao Y (2014). Siegenite: A New Gold-Bearing Mineral. Acta Geologica Sinica..

[25] Ma, F. *Study on the Enrichment Regularity of Mineralization of Jinchang Gold Deposit*, *Dongning in Heilongjiang Province*: (JiLin University, 2014).

[26] Sillitoe RH (2010). Porphyry Copper Systems. Econ Geol..

[27] Zhang W (2008). Fluid Inclusion Indicators in Prophyry Au Deposits: Taking Jinchang Gold Deposit, Heilongjiang Province as an Example. Acta Petrol Sin..

[28] Lee J (2006). A Redetermination of the Isotopic Abundances of Atmospheric Ar. Geochim Cosmochim Ac..

[29] Kelley SP, Turner G, Butterfield AW, Shepherd TJ (1986). The Source and Significance of Argon Isotopes in Fluid Inclusions From Areas of Rnineralization. Earth Planet Sc Lett..

[30] Kendrick MA, Burgess R, Pattrick RAD, Turner G (2001). Halogen and Ar-Ar Age Determinations of Inclusions within Quartz Veins From Porphyry Copper Deposits Using Complementary Noble Gas Extraction Techniques. Chem Geol..

[31] Ren Y (2016). The Age, Geological Setting, and Types of Gold Deposits in the Yanbian and Adjacent Areas, NE China. Ore Geol Rev..

[32] Piquer J, Cooke D, Chen J, Zhang L (2017). Synextensional Emplacement of Porphyry Cu-Mo and Epithermal Mineralization: The Zijinshan District, Southeastern China. Econ Geol..

[33] Xiao W, Windley B, Hao J, Zhai M (2003). Accretion Leading to Collision and the Permian Solonker Suture, Inner Mongolia, China: Termination of the Centr. Tectonics..

[34] Wilde SA, Zhou J (2015). The Late Paleozoic to Mesozoic Evolution of the Eastern Margin of the Central Asian Orogenic Belt in China. J Asian Earth Sci..

[35] Xu W (2013). Spatial–Temporal Relationships of Mesozoic Volcanic Rocks in NE China: Constraints On Tectonic Overprinting and Transformations Between Multiple Tectonic Regimes. J Asian Earth Sci..

[36] Zhang J (2010). Geochronology of the Mesozoic Volcanic Rocks in the Great Xing’an Range, Northeastern China: Implications for Subduction-Induced Delamination. Chem Geol..

[37] Wu F, Sun D, Li H, Jahn B, Wilde S (2002). A-Type Granites in Northeastern China: Age and Geochemical Constraints On their Petrogenesis. Chem Geol..

[38] Liu J, Davis GA, Lin Z, Wu F (2005). The Liaonan Metamorphic Core Complex, Southeastern Liaoning Province, North China: A Likely Contributor to Cretaceous Rotation of Eastern Liaoning, Korea and Contiguous Areas. Tectonophysics..

[39] Yang J (2007). Rapid Exhumation and Cooling of the Liaonan Metamorphic Core Complex: Inferences From ^40^Ar/^39^Ar Thermochronology and Implications for Late Mesozoic Extension in the Eastern North China Craton. Geol Soc Am Bull..

[40] Zhang J, Ge W, Wu F, Liu X (2006). Mesozoic Bimodal Volcanic Suite in Zhalantun of the Da Hinggan Range and its Geological Significance: Zircon U-Pb Age and Hf Isotopic Constraints. Acta Geol Sin-Engl..

[41] Meng Q (2003). What Drove Late Mesozoic Extension of the Northern China–Mongolia Tract?. Tectonophysics..

[42] Deng J, Wang C, Bagas L, Carranza EJM, Lu Y (2015). Cretaceous–Cenozoic Tectonic History of the Jiaojia Fault and Gold Mineralization in the Jiaodong Peninsula, China: Constraints From Zircon U-Pb, Illite K-Ar, and Apatite Fission Track Thermochronometry. Miner Deposita..

[43] Sun W (2013). Large-Scale Gold Mineralization in Eastern China Induced by an Early Cretaceous Clockwise Change in Pacific Plate Motions. Int Geol Rev..

[44] Goldfarb RJ, Hart C, Davis G, Groves D (2007). East Asian Gold: Deciphering the Anomaly of Phanerozoic Gold in Precambrian Cratons. Econ Geol..

[45] Choi SG, Kwon ST, Ree JH, So CS, Pak SJ (2005). Origin of Mesozoic Gold Mineralization in South Korea. The Island Arc..

[46] Maruyama S, Send T (1986). Orogeny and Relative Plate Motions: Example of the Japanese Islands. Tectonophysics..

[47] Zhang Z (2010). Geochemistry and Geochronology of the Volcanic Rocks Associated with the Dong’an Adularia–Sericite Epithermal Gold Deposit, Lesser Hinggan Range, Heilongjiang Province, NE China: Constraints On the Metallogenesis. Ore Geol Rev..

[48] Wang S (1983). Age determinations of ^40^Ar-^40^K, ^40^Ar-^39^Ar and radiogenic ^40^Ar released characteristics on K-Ar geostandards of China. Sci. Geol. Sin..

[49] Qiu H, Bai X, Liu W, Mei L (2015). Automatic ^40^Ar/^39^Ar Dating Technique Using Multicollector ArgusVI MS with Home-Made Apparatus. Geochimica..

[50] Koppers AAP (2002). ArArCALCFsoftware for ^40^Ar/^39^Ar Age Calculations. Comput Geosci-Uk..

